# From Parental Behavior to Sexual Function: Recent Advances in Oxytocin Research

**DOI:** 10.1007/s11930-024-00386-1

**Published:** 2024-05-11

**Authors:** Joseph Dale II, Mitchell T. Harberson, Jennifer W. Hill

**Affiliations:** 1 Center for Diabetes and Endocrine Research, University of Toledo College of Medicine, Toledo, OH USA; 2 Department of Physiology and Pharmacology, University of Toledo College of Medicine, Toledo, OH USA; 3 Department of Obstetrics and Gynecology, University of Toledo College of Medicine, Toledo, OH USA; 4 Department of Biology, University of Toledo College of Medicine, Toledo, OH USA

**Keywords:** Oxytocin, Paraventricular Hypothalamic Nucleus, Social Behavior, Sexual Behavior, Maternal Behavior, Neuroanatomy

## Abstract

**Purpose of Review:**

Oxytocin plays many diverse roles in physiological and behavioral processes, including social activity, parental nurturing, stress responses, and sexual function. In this narrative review, we provide an update on the most noteworthy recent findings in this fascinating field.

**Recent Findings:**

The development of techniques such as serial two-photon tomography and fiber photometry have provided a window into oxytocin neuroanatomy and real-time neuronal activity during social interactions. fMRI and complementary mapping techniques offer new insights into oxytocin's influence on brain activity and connectivity. Indeed, oxytocin has recently been found to influence the acquisition of maternal care behaviors and to mediate the influence of social touch on brain development and social interaction. Additionally, oxytocin plays a crucial role in male sexual function, affecting erectile activity and ejaculation, while its role in females remains controversial. Recent studies also highlight oxytocin's interaction with other neuropeptides, such as melanin-concentrating hormone, serotonin, and arginine vasopressin, influencing social and affective behaviors. Finally, an update is provided on the status of clinical trials involving oxytocin as a therapeutic intervention.

**Summary:**

The exploration of oxytocin's complexities and its interplay with other neuropeptides holds promise for targeted treatment in various health and disease contexts. Overall, these findings contribute to the discovery of new and specific pathways to allow therapeutic targeting of oxytocin to treat disorders.

## Introduction

Oxytocin is a well-known and highly-studied nonapeptide hormone produced by neurons in the supraoptic nucleus (SON) and the paraventricular nucleus of the hypothalamus (PVH). Magnocellular oxytocin neurons in these areas project to the posterior pituitary gland where they release oxytocin into circulation [1]. The most well-known function of oxytocin is inducing contractions in the uterus during labor and in the breast tissue during breast feeding. Oxytocin can also be released centrally in the brain to modulate the activity of neurons and neural circuits to regulate a range of social and non-social processes. Oxytocin research has primarily focused on its regulation of social behaviors such as parental nurturing, pair bonding, partner preference, sensory processing, empathy, and sexual function [2, 3]. How oxytocin modifies such a large range of social behaviors is a major open question. One of the dominant hypotheses, the “social salience” hypothesis, claims that oxytocin does not promote prosocial behaviors but rather increases the salience of social stimuli by acting on the mesolimbic dopamine system[4]. The primary evidence for this hypothesis is that oxytocin action is context-specific rather than unidirectional in its actions. For example, oxytocin increases love, trust, and empathy only toward in-group members while promoting aggression toward out-group members[5, 6]. Inconsistencies in the literature are also explained by oxytocin action being sex-specific[7, 8], species-specific[9, 10], and specific to the stage of development[11]. Despite this complexity, scientists have used advances in genetic, molecular, and anatomical biology to help understand the underlying mechanisms controlling behaviors. As detailed below, these studies represent a major advance in our understanding of oxytocin.

## New Neuroanatomical Insights

New advances in genetic models, technology, and computer software [12] have enhanced our understanding of oxytocinergic neuroanatomy, activity, and interactivity with other neuronal cell types. Recently, Son and colleagues have used whole-brain mapping techniques to visualize oxytocin's distribution, projections, and overlap with oxytocin receptor (OXTR) expression in mice [13••]. The largest clusters of oxytocin neurons were found in the PVH, SON, accessory nuclei (AN), and the often-overlooked tuberal nucleus (TU), which had nearly as many neurons as the PVH. Imaging the separate projections of these four nuclei yielded data that is well-supported by other recent publications [14, 15]. The PVH projected widely to nine functional circuits; the AN and SON may regulate these circuits through their projections to the PVH. The TU had no long-range projections. Interestingly, most projection areas had reciprocal connections with oxytocinergic circuits. Although most oxytocin neurons project to midbrain and hindbrain regions, OXTR is expressed primarily in cortical regions. Indeed, when the oxytocin projectome data were compared to whole-brain OXTR expression, no correlation was found except in the thalamus and medulla. Furthermore, this publication found oxytocin fibers contacting the surface of the lateral, third, and fourth ventricles [13••]. Taken together, these results support the well-documented hypothesis that oxytocin action is partially mediated through transmission into the cerebrospinal fluid (CSF) [16]. More specifically, oxytocin secretion into the CSF of the lateral ventricle has been found to be important for its actions in the cerebral cortex[17]. This mechanism may explain how oxytocin interacts with the high cortical expression of OXTR found by Son and colleagues. However, other studies have found most regions that express OXTR have at least a small number of oxytocinergic projections[9]; therefore, oxytocin action is likely mediated through both CSF and direct transmission. The non-ventricular oxytocin projections that seemingly have no local OXTR expression could be instances of oxytocin acting on other receptors such as vasopressin receptors[18] or TRPV1 receptors[19]. An interesting alternative hypothesis was proposed by Grinevich and coworkers that states these oxytocinergic projections may be secreting glutamate from these synapses while secreting oxytocin non-synaptically (i.e. somatodendritically)[20]. Indeed, evidence suggests the fear-related behavioral effects of oxytocin neurons are mediated through synaptic glutamate, not oxytocin[21].

Zhang and colleagues recently used serial sectioning tomography to image magnocellular oxytocin neurons [22] at a higher resolution than previously performed [23]. Excluding their pituitary projections, these neurons project to cortical (piriform cortex, auditory cortex) and subcortical (amygdala, nucleus accumbens, caudoputamen, lateral septum) regions. When these magnocellular neurons were activated or inhibited, they increased or decreased social investigation and interaction, respectively [22]. These findings are reinforced by another study using tissue clearing to image oxytocin and vasopressin populations in the developing mouse brain [24]. Overall, these studies provide a clear picture of the major oxytocin circuits that may regulate attention, threat detection, sleep/wake, pain, sensory motor regulation, metabolism, learning, memory, reward, and reproductive functions. In future studies, these mapping techniques could be used to image whole-brain activation levels of oxytocin neurons using the expression of c-fos; for example, one study imaged neural activation during social recognition [25].

One of the major drawbacks of whole-brain mapping techniques is that they only capture a snapshot of neuroanatomy and neural activity. Therefore, these studies are nicely complemented by fMRI in mice, which can capture whole-brain dynamic changes in activity and connectivity [12]. For example, one recent study, which used both pharmacological MRI and tissue clearing with c-fos staining, found differences in brain activity in the Cntnap2 KO mouse model of autism spectrum disorder [26]. Differences in functional connectivity were also found using resting-state MRI. Interestingly, deficits in brain activity and connectivity were reversed after i.p. injection or endogenous release of oxytocin. The nucleus accumbens (NAc) was one such region that saw this reversal in brain activity and connectivity, so it was further studied for its role in social salience and reward. Injection of an oxytocin agonist into the NAc or activation of oxytocin axons in the NAc increased social interaction time in Cntnap2 KO mice, showing oxytocin action in the NAc promotes sociality in this context [26]. Overall, whole-brain activation and connectivity studies have further reiterated the importance of oxytocin action in the nucleus accumbens, which further bolsters the “social salience” hypothesis.

One of the most powerful imaging techniques for mechanistic studies is fiber photometry, or the use of an optic fiber to measure fluorescence as an indicator of activity. For example, genetically encoded indicators of calcium—such as the GCaMP6 proteins—fluoresce during neuronal activation and offer real-time tracking of activity in mice. Tang and colleagues tracked oxytocin neuronal activity during social interaction in freely moving mice using fiber photometry and tetrode measurements [27•]. Social touch activated oxytocin neurons to the greatest extent when the mouse was either crawling on top of another animal or being crawled on. Interestingly, social touch increased c-fos in parvocellular oxytocin neurons but not magnocellular oxytocin neurons. The parvocellular neurons in the PVH were previously found by the authors to project to the SON and act on magnocellular oxytocin neurons to mediate nociception [28]. Using chemogenetics, activation of parvocellular oxytocin neurons projecting to the SON increased the free social interactions in mice and activity in magnocellular neurons, while inhibition had the opposite effect. These results suggest social touch releases oxytocin by first activating parvocellular oxytocin neurons in the PVH, which then activate magnocellular oxytocin neurons in the SON [27•]. Another study tracked the activity of hundreds of individual PVH oxytocin neurons in head-fixed mice by imaging GCaMP6 via two-photon microscopy [29]. Two-photon microscopes are known for superior tissue penetration yet are not powerful enough to reach the hypothalamus; thus, the authors used the cutting-edge approach of mounting a micro gradient refractive index lens (GRIN) to the top of the mouse head to improve tissue penetration. When exposed to a social stimulus (a juvenile mouse), a greater proportion of oxytocin neurons were activated, and a lower proportion of oxytocin neurons were inhibited compared to a non-social stimulus (a toy mouse). Subpopulations of oxytocin neurons were activated by both stimuli or had opposing responses to both stimuli—for example, being activated by social stimuli and inactivated by non-social stimuli. Interestingly, the subpopulations with opposing responses had low spontaneous activity while populations activated by both stimuli had high spontaneous activity. These results suggest a subpopulation of PVH oxytocin neurons with low spontaneous activity responds to social stimuli [29]. Overall, real-time tracking of oxytocin neuronal activity using fiber photometry has improved our understanding of how oxytocin neurons are activated and how their activation regulates social behavior. Future studies could use fluorescence miniscopes mounted above the GRIN lens to track hundreds of oxytocin neurons in freely moving animals [12, 30, 31]. Other novel technologies such as a genetically encoded oxytocin sensors are also being rapidly adopted for pathway analysis [32, 33].

## Recently Identified Neuropeptide Interactions

New oxytocin research has provided insight into the dynamic interactions of this peptide with other neuropeptide pathways. Among these, the interplay between oxytocin, melanin-concentrating hormone (MCH), serotonin, and vasopressin is highlighted in recent studies on social and affective behaviors.

MCH is a peptide neurotransmitter produced in the lateral hypothalamus and zona incerta that is perhaps best known for promoting appetitive behaviors and regulating arousal [34]. Like oxytocin, MCH regulates sexual [35], social [36], and maternal behaviors [37, 38], and has recently been found to be downstream of oxytocin signaling [39]. Indeed, MCH neurons receive oxytocinergic inputs from the PVH, express OXTR on nearly 60% of cells[39], and respond to oxytocin with increased activity [40]. Recently, oxytocin's effects on repetitive behaviors and social recognition have been found to be mediated by MCH. Sanathara and colleagues found that oxytocin or MCH treatment of male mice reduced marble burying, a measure of repetitive behavior. No additive effect was seen when oxytocin and MCH were given together. Further, the oxytocin-driven increase in marble burying was blocked by co-treatment with an MCH receptor antagonist. Another study found mice have impaired social recognition when OXTRs were knocked out only in MCH neurons. While wild-type (WT) mice spent more time with novel mice, OXTR-MCHKO mice spent an equal amount of time with familiar and novel mice [41]. A similar OXTR-MCHKO model was used to determine that oxytocin signaling on MCH neurons regulates mood and depressive behavior [42]. Taken together, these results raise the possibility that some of oxytocin's therapeutic effects on symptoms of autism spectrum disorder—including reduced repetitive behaviors and improved social recognition [39, 41]—are mediated by MCH.

Serotonin is also involved in the effects of oxytocin on anxiety and social reward. Serotonin is widely expressed in the raphe nuclei (RN). The RN receives input from PVH oxytocin neurons [13••, 43] and expresses OXTR in rodents [44]. Oxytocin increases the availability of serotonin receptors in the amygdala, insula, and hippocampus in primates and humans and induces serotonin release in those same regions in primates [45, 46]. Yoshida and colleagues found that oxytocin treatment promoted the release of serotonin in the median raphe nucleus and increased the time spent in the center of an open-field test, indicating less anxiety. The decrease in anxiety-like behavior was blocked when oxytocin was co-administered with an OXTR antagonist [44]. Another study found that oxytocin can mediate social reward by inducing neuroplastic changes in the caudal nucleus accumbens. This effect depended on oxytocin signaling in serotonin neurons in the dorsal raphe [47]. While these studies clarify that oxytocin regulates serotonin neurons, one recent paper found the converse; serotonin regulates oxytocin neuron activity when a mouse pup first interacts with its mother [48•]. Maternal odors not only activated oxytocin neurons in the infant but serotonin neurons in the RN as well. Mice lacking the tryptophan hydroxylase 2 enzyme, necessary for serotonin synthesis, showed impaired maternal affiliation, which was restored by oxytocin treatment. Lastly, these deficits were also rescued by the activation of oxytocin neurons in the PVH or serotonergic projections to the PVH [48•]. Overall, these results suggest that the reciprocal connections between RN serotonin neurons and PVH oxytocin neurons influence anxiety, social reward, and maternal affiliation.

Vasopressin and oxytocin evolved from the same precursor gene that was duplicated over 600 million years ago and share seven out of nine amino acids [18]. Further, the OXTR has 85% sequence homology with the most prominent vasopressin receptor (V1aR). Indeed, crosstalk between these hormones has been discovered [18, 49]. Peripheral effects of oxytocin, such as reducing heart rate and body temperature [50] and inducing ejaculatory contractions, are blocked by V1aR but not OXTR antagonists [18]. In addition, the central effects of oxytocin, such as induction of flank marking (a rodent dominance behavior where odors are secreted from the flank gland) are blocked by V1aR antagonists [18]. The extent to which this crosstalk influences study results, endogenous hormone action, or exogenous hormone medications is an area of recent interest to the field. An ex vivo study found that both hormones have the highest affinities for their cognate receptors; in fact, oxytocin had a 100-fold higher affinity for OXTR over V1aR, and vasopressin had a sevenfold higher affinity for V1aR over OXTR [51]. The crosstalk in this study is less than previously reported [18] and has been supported by follow-up studies in other species[52]. Another set of studies found consistent crosstalk among receptors from different primate species and their oxytocin variants, but vasopressin showed the highest efficiency and potency for V1aR activation [52, 53]. Lastly, one study compared oxytocin and vasopressin fiber density with V1aR and OXTR binding densities in key brain regions in the rat. The medial amygdala, bed nucleus of the stria terminalis (BNST), mPOA, and periaqueductal gray (PAG) stood out as the nuclei with the greatest overlap and therefore were concluded to be most susceptible to crosstalk [54]. Similar interactions between the oxytocin and vasopressin systems may occur peripherally in pregnant women with elevated oxytocin levels [55]. Thus, when studying oxytocin, the possibility of V1aR activation should be considered. For example, endogenous oxytocin can still activate V1aR in OXTR KO animals, so models with both OXTR KO and endogenous oxytocin blockade may be valuable for further understanding this crosstalk. Action at vasopressin receptors must also be considered when analyzing the effects of medications that can raise oxytocin to hyper-physiological levels [56, 57]. However, the exact magnitude of crosstalk will be difficult to quantify until consistent methods for measuring oxytocin centrally and peripherally can be established [58].

## New Insights into Maternal Behavior

Oxytocin triggers maternal behaviors, including the ability of a mouse dam to recognize and respond to the calls of her pups [59]. In mice, oxytocin enables maternal recognition of pup distress by acting in the auditory cortex. The Froemke lab has shown a high concentration of oxytocin neuronal projections in the left auditory cortex [60, 61]. They found dams receiving oxytocin in the left auditory cortex showed pup retrieval and maternal behaviors earlier than saline-infused dams. Similar results were found with optogenetic stimulation of the same region. Activity in these oxytocin neurons led to changes in auditory cortex firing in response to pup calls, which allowed mice to recognize pup calls and facilitated maternal care.

Building on this research, Valtcheva and colleagues have recently used chemogenetics to show that oxytocin allows dams to learn how to respond to pup auditory calls [62]. They monitored the ability of virgin mice to show alloparenting, which is providing parental care to pups that are not their own. Virgin mice co-housed with an experienced dam and pups responded to pup auditory calls roughly 50% quicker than virgin females housed with pups alone. This finding shows the importance of social learning, whereby the experienced dam demonstrated appropriate responses to pup calls for the virgin female. Interestingly, pup retrieval in virgin and experienced mice triggered increased activity in oxytocin neurons. The researchers then compared normal mice and Oxy-cre mice injected with an inhibitory designer receptor exclusively activated by designer drugs (DREADD) to suppress the activity of oxytocin neurons in the PVH. When oxytocin neurons were inactivated, virgin females did not effectively respond to pup vocal signals over a 4-day testing period even when housed with an experienced dam. Thus, social learning of alloparenting requires functional oxytocinergic neurons in the PVH. Since the females had physical contact with the pups during their calls, the studies did not test if the auditory signal of the pups alone triggered oxytocin signaling. It is therefore unclear whether the oxytocin signaling modulating parental behavior was activated through touch or sound.

Social touch is essential for normal mammalian brain development in young organisms. Harry Harlow established the importance of social touch in his classic studies showing young macaques preferred spending time with surrogate mothers made of soft terrycloth to wire ones that provided food [63]. Further, monkeys lacking maternal touch exhibited anxiety-like behaviors and did not interact normally with their peers [64]. In humans, early positive somatosensory interaction such as cuddling, breastfeeding, and exposure to the scent of their parents is linked to a decreased chance of adolescent anxiety disorders [65]. A recent study in mice used a machine to mimic maternal touch and licking to examine the effects of tactile touch on juveniles [65]. Social touch increased the spontaneous firing rate of oxytocin neurons in acute ex vivo slices. Oxytocin firing induced by tactile touch also promoted increased social interaction in a substance P-dependent manner. Indeed, a group of periaqueductal gray substance P-producing, Tac1-expressing neurons known primarily for their role in nociception [66••] were found to send monosynaptic excitatory projections to the PVH. Social touch increased their firing rates, while their activation induced social interaction. The authors propose these neurons convey the experience of pleasant social touch via oxytocin neuron activation to elicit social behavior [65]. Overall, these studies serve as an important stepping-stone in the development and use of oxytocin for clinical conditions that cause social deficiencies. Indeed, a clinical trial, described later, has also recently investigated the role of oxytocin in human social touch.

## Advances in the Sexual Function Field

Oxytocin's neurophysiological influence extends to a variety of well-established roles, including male sexual physiology and function. These actions involve communication with key brain regions, such as the BNST and PVH. Recent studies have reinforced the importance of oxytocin in male sexual function and interrogated the sexual dimorphism of this system.

In the 1980s, Argiolas and colleagues found that intracerebroventricular injections of oxytocin induced penile erections in rats [67]. Later studies showed administering oxytocin to the medial preoptic area (mPOA), PVH [68], ventral tegmental area [69•], or the BNST[70] elicited erectile effects in mice and triggered c-fos expression in these regions. OXTR antagonists blocked the effect of direct oxytocin injection into the BNST, potentially by acting on glutamatergic neurons [8, 68, 69•, 71]. Activation of the OXTR in the PVH leads to an influx of calcium and activation of nitric oxide synthase in the brain that promotes erectile activity [8, 68, 69•, 71]. Another proposed pathway for the erectile effects of oxytocin involves the activation of serotoninergic lumbosacral spinal neurons that send signals directly to the pudendal nerves facilitating penile erection. This mechanism is supported by findings showing internasal administration of oxytocin in males increases 5-HT receptor activity in the lumbosacral spinal cord [46, 72, 73]. Oxytocin may not be essential for normal erectile function and sexual function in male rats, however, because rats lacking the oxytocin gene show normal sexual activity and receptivity [69•, 74]. Alternatively, its functional importance may be masked by the functional redundancy of other neuropeptides in this model [75].

Premature ejaculation is the most common cause of male sexual dysfunction, affecting up to 30% of men worldwide[76]. Lumbar spinothalamic cells that trigger ejaculation in rats send signals to and receive them from hypothalamic oxytocin neurons [68]. In addition, peripheral effects of oxytocin on ejaculation have been hypothesized based on data showing the OXTR is present in the human epididymis and mediates contractile activity necessary for seminal release [77]. Recently, Beatrix Stadler and colleagues showed that oxytocin elicited a large concentration-dependent tonic response in the muscle of the corpus spongiosum, bladder neck, and prostate urethra [78]. In ex vivo preparations of these tissues treated with a strong OXTR antagonist, oxytocin could still elicit a tonic effect in penile tissue. The researchers found this action required oxytocin binding to the vasopressin receptor subtype V1B in these tissues. Others have found that oxytocin administered systemically reduced the time taken for the first mount, intromission, and ejaculation in males [79]. These findings suggest systemic oxytocin signaling can improve ejaculation efficiency [15, 78].

Some oxytocin neurons in the PVH express the melanocortin 4 receptor (MC4R) [80], whose activation can stimulate oxytocin release [81]. Notably, Peñagarikano and co-authors (2015) discovered that inducing the release of oxytocin with a specific MC4R agonist alleviated social deficits in the *Ctnap2* transgenic mice [82]. Thus, inducing oxytocin release via exogenous drugs can mimic the actions of endogenous oxytocin in mice. New data from our lab suggest MC4R activation also leads to oxytocin neuron activation during ejaculation [83]. Transgenic mice lacking the MC4R in all cells showed delayed ejaculation compared to WT mice. This phenotype was not seen in mice with the MC4R expressed only on oxytocin neurons, demonstrating sufficiency of oxytocin neurons for mediating this effect. Importantly, the groups were matched at young ages to control for the effects of obesity. Thus, MC4R-mediated melanocortin signaling on oxytocin neurons promotes normal ejaculation independent of the male's metabolic status. These studies demonstrate that melanocortin-responsive oxytocin pathways are sufficient for ejaculatory function, but do not rule out the existence of additional, redundant pathways.

A comprehensive understanding of oxytocin's involvement in sexual function will provide potential avenues for therapeutic advances for conditions such as erectile and ejaculatory dysfunction. Central treatments for erectile function are not currently being pursued but could be an avenue for treatment when psychogenic erectile dysfunction is suspected [84]. Unraveling the intricacies of this neurocircuitry will pave the way for better treatment choices for men dealing with sexual dysfunction.

Oxytocin may facilitate female sexual function and receptivity as well. In female rodents, mating increases c-fos expression in oxytocin neurons [85] and oxytocin levels in the PVH [86]. Administration of oxytocin into either the medial preoptic area (mPOA) or the ventromedial hypothalamus (VMH) improves sexual receptivity [87, 88]. Female mice lacking oxytocin show decreased, but not eliminated, lordosis behavior [89, 90]. In prairie voles, oxytocin increases sexual receptivity in sexually naïve but not experienced females [91, 92].

Whether these results translate to humans is unclear. Oxytocin has the potential to improve sexual function in women, in that increased blood oxytocin levels accompany sexual arousal and orgasm [93]. However, intranasal oxytocin was recently found to have no effect on sexual drive, arousal, or orgasm during self-stimulation in a laboratory setting, nor did it influence physiological parameters such as vaginal blood volume [94]. However, a similar study where participants had sex with their partners at home found that women were more relaxed, better at communicating their sexual desires, and better at empathizing with their partner after intranasal oxytocin treatment [95]. Here is yet another example of oxytocin promoting social salience of a stimulus. Thus, oxytocin in women may not affect sexual desire, arousal, or orgasm, but instead enhance overall experience by promoting partner bonding and the social benefits of sex. This hypothesis is supported by the role of mating-induced oxytocin release in the formation of pair bonds in monogamous species [96] and the importance of oxytocin release in the formation and maintenance of romantic relationships in humans [97, 98].

## Translating Oxytocin Research from Bench to Bedside

As we have seen, mechanistic studies of oxytocin action on brain pathways in animal models have advanced substantially with new techniques to manipulate and measure neuronal activity. These approaches have increased our understanding of the pathways involved in the behavioral effects of centrally administered oxytocin in animals. The encouraging behavioral effects of oxytocin in animals have captured widespread interest. Many are intrigued by the clinical potential of oxytocin as a treatment for psychopathologies related to socio-emotional dysfunction, including autism spectrum disorders, anxiety disorders, social phobia, and schizophrenia [99–101]. Human research relies on non-invasive approaches like observational studies on oxytocin and social behavior, translational studies on patients with social behavior disorders, and intervention studies with peripheral oxytocin administration. Clinical trials exploring the administration of oxytocin have yielded mixed results, and the effectiveness of oxytocin in various contexts is still an area of ongoing research [102–104]. Several different approaches have been taken to leverage the power of oxytocin in clinical applications.

As intracerebral administration is impractical in humans, researchers have turned to intranasal administration to manipulate oxytocin levels in the brain. The application of this method assumes that when oxytocin is administered intranasally, it raises oxytocin levels in the central nervous system through nose-to-brain pathways. Intranasal administration is believed to induce psychological and behavioral effects by engaging central oxytocin receptors. The advantages of this pain-free, non-invasive, and direct administration are clear. However, the absence of observable effects in both healthy and clinical populations following intranasal oxytocin administration [105–108] has prompted consideration about the reliability of earlier reported positive effects [102, 103, 109]. One important finding in translational/clinical research on oxytocin in recent years was the unsuccessful phase II study using intranasal oxytocin to address social behavioral deficits in autism [104]. A notable critique of this trial was that, based on the social salience hypothesis, oxytocin would not be expected to have a therapeutic impact unless it was administered within a therapeutic context [110]. Indeed, a preliminary study discovered that intranasal oxytocin was beneficial, but only when administered alongside a positive social environment (as an adjunct to therapy) [111]. More recently, a study found that long-term intranasal oxytocin administration triggered the body's natural oxytocin system [112]. This research suggests that combining oxytocin treatment with social interaction (such as psychosocial therapy) could enhance the body's natural oxytocin feedback loop.

Despite extensive research, the question of whether intranasal oxytocin administration is capable of accessing the brain has not been resolved [113]. For example, intranasal administration in oxytocin knockout mice, who cannot produce oxytocin, increased levels in brain tissue [114]. While peripheral oxytocin appears to reach the brain via transport across the blood–brain barrier (BBB) in both rodents and primates [57, 115], only a small percentage of administered oxytocin crosses the barrier into the CSF (up to 0.005%) even when given at supraphysiological levels [113]. Moreover, CSF measures of oxytocin may not represent oxytocin's action within brain tissue, where oxytocin receptors are located. An alternative route of brain penetration for intranasal oxytocin is convective bulk transport in the CSF that flows within the perivascular spaces around the olfactory and trigeminal nerves [116, 117]. Recent research in rats showed increased detection of an oxytocin receptor radiotracer in the trigeminal and olfactory nerves and olfactory bulbs of rats after intranasal administration compared to intravenous administration [118]. Uptake into the brain via circumventricular organs lacking the BBB but expressing OXTR requires further investigation [119]. Only a single human study with a small sample size found increased oxytocin levels in CSF following intranasal oxytocin administration [120]. Additional study of the brain penetrance of intranasal oxytocin in humans is critical for determining its value as a therapeutic [121]. Looking ahead, exploring the development of oxytocin agonists or antagonists capable of crossing the BBB may offer a better alternative than intranasal administration.

Dysregulation of oxytocin has also been hypothesized to play a role in the pathophysiology of schizophrenia. Goh and colleagues hypothesize that decreased oxytocin dysregulates reward signaling and improperly assigns emotional salience, which leads to paranoia and social isolation [122]. In support of this hypothesis, several studies have found both positive and negative symptoms of schizophrenia negatively correlate with serum oxytocin levels [122]. Many studies have tested oxytocin as a treatment for schizophrenia alongside antipsychotics. Although some studies have found oxytocin improves the positive and negative symptoms of schizophrenia, other studies have found no significant difference from placebo [122]. Separately, oxytocin could also be relevant for the treatment of post-traumatic stress disorder. MDMA has been found to improve symptoms for post-traumatic stress disorder (PTSD), these positive effects have been speculated to be mediated through oxytocin signaling [123]. Because the oxytocin system is involved in a wide array of neural processes, it remains an interesting potential therapeutic for a multitude of neuropsychiatric conditions.

Interestingly, a clinical trial called "The Effects of Oxytocin Treatment on Social Touch" led by Dr. Qin Li, of the University of Electronic Science and Technology of China has recently investigated the use of oxytocin to increase the pleasure experienced during social touch in humans [48•]. The study was done in a randomized manner with placebo drug controls. The trial specifically sought to determine the best administration route for oxytocin, oral or intranasal, to affect pleasure perceived by gentle touch. Touch sensations activate different nerve fibers depending on the strength of skin contact, leading to the activation of separate sensory pathways. They therefore sought to determine if oral or intranasal treatment affected the subjective experience of gentle vs. forceful touch during massage therapy. They found that both administration routes increased the plasma concentration of oxytocin and the perceived pleasantness of the gentle massage. The perception of the forceful massage was not altered [48•]. The study did not explain if a central or peripheral action of oxytocin drove the effects they observed, and a potential mechanism of action for oral oxytocin is lacking [124]. OXTRs are widely present in peripheral organs like the heart, skin, intestinal tract, and autonomic nervous system. Peripheral oxytocin has numerous effects [125–127]. For instance, peripheral oxytocin increases insulin and glucagon secretion [128], regulates gastric motility [129], activates vagal afferent neurons [130], and increases heart rate variability [131]. Oxytocin action in the periphery is often linked to its central action; for example, oxytocin promotes birth and breast feeding peripherally but also maternal behaviors centrally [15]. Thus, peripheral oxytocin may regulate perception of social touch through actions on peripheral organs [119]. This suggestion is supported by the aforementioned studies that found social touch activates oxytocin neurons in the PVH [132] and is in accordance with the social salience hypothesis that states central oxytocin increases salience for social stimuli. Lastly, social touch is valuable for human development, the formation of relationships, and social reward; thus, enhancing social touch in individuals with autism spectrum disorder (ASD) could provide another therapeutic approach [133].

Clinical trials studying oxytocin are likely to continue and advance to other areas of disease treatment as our understanding of its pharmacological effects strengthens. To date, clinical studies have not found oxytocin toxicity or any major side effects after administration, regardless of the dosages or routes of administration used (IV, Oral, Intranasal) [134, 135]. It is possible, however, that when oxytocin is administered over an extended period, it may lead to adverse effects. In rodents, chronic oxytocin treatment is associated with changes in social behaviors and the expression of OXTRs within the brain [136–139]. Additionally, individuals with pre-existing heart conditions might be at a higher risk of experiencing negative effects from the cardiovascular actions of oxytocin [134, 140, 141].

Overall, current evidence regarding the effect of exogenous oxytocin on human social behavior has been judged to be weak [142]. Several specific concerns have been raised regarding this body of research in humans, including the heterogeneity of findings, the lack of replication of results, and the need for detailed dose–response studies [143]. In addition, approaches used were exploratory and critically underpowered, with oxytocin randomized controlled trials reported as having 16% statistical power on average [143]. There is a clear need to calculate precise sample size estimates when planning studies [144, 145]. Recent articles advise the use of standardized methods and study pre-registration to improve oxytocin measurement precision [58, 113, 145]. These steps may help the field overcome problems with methods and reproducibility and lead to a clear picture of how oxytocin affects human behavior.

An alternative to exogenous oxytocin administration is inducing endogenous oxytocin release within established neural circuits. In particular, melanocortin receptor agonists hold great potential for stimulating the endogenous oxytocin system, as previously mentioned. Oxytocin neurons in the PVH and SON express MC4R [80] and are activated by MC4R agonists [81]. Both central and peripheral administration of MC4R agonists increases oxytocin levels in the hypothalamus [146, 147]. One of the most recent advances in female sexual health was the FDA approval of the MC4R agonist bremelanotide to treat hypoactive sexual desire disorder. Not only do data in rats support bremelanotide's efficacy [148], but bremelanotide's effects may be partially mediated by oxytocin neurons. Recently, our lab found that knock out of MC4R reduced sexual receptivity in female mice independent of body weight [80]. Interestingly, expression of MC4R only in Sim1 neurons—which are prominently found in the PVH—rescued sexual receptivity. However, when MC4R was only expressed in oxytocin neurons—which are a subset of Sim1 neurons—sexual receptivity was not fully normalized [80]. Therefore, oxytocin mediates some but not all the pro-sexual effects of melanocortins in female mice. The sexually dimorphic roles of the oxytocin and melanocortin systems in sexual behavior and function deserve further study.

In accordance with the capacity for melanocortins to induce oxytocin release, preclinical studies have found melanocortin treatment improves social behavior in rodent models with social deficits. First, in a maternal immune activation mouse model of ASD, melanocortin treatment over 7 days improved their sociability index to the same level as WT mice[149]. Second, the CNTNAP2 KO mouse model of ASD had a full recovery in social interaction after treatment with a melanocortin agonist[82]. Third, a neonatal isolation model in prairie voles decreased time spent with their partner in a preference test; preference for their partner was restored after treatment with melanocortins[150]. Interestingly, similar to oxytocin, the melanocortin-induced brain activation is different in social vs non-social contexts and is affected by OXTR antagonists[151]. Overall, melanocortins are brain penetrant, safety-tested drugs that could be used for treatment of ASD due to their action on oxytocin neurons.

## Conclusions

In conclusion, oxytocin research continues to uncover new roles for this peptide in diverse biological and behavioral processes. From its classical functions in labor and lactation to its involvement in social behaviors, parenting, and sexual functions, oxytocin is a central player in shaping mammalian physiology. Advances in genetic models, neuroanatomy mapping, and innovative technologies have provided unprecedented insights into the spatiotemporal regulation of oxytocin and its dynamic interactions with other neuropeptide pathways. Notably, research into oxytocin's interplay with MCH, serotonin, and vasopressin offers valuable perspectives on shared regulatory controls over social and affective behaviors. These advances in oxytocin provide support for the social salience hypothesis and help to tease apart the complex web of oxytocin’s interactions. These advances also pave the way for potential therapeutic interventions. The increasing array of techniques and resources available, along with advancements in translational research [187, 188] may allow the quality of study results to continue to improve in the coming years. Despite recent studies that found no effect of intranasal oxytocin treatment in adolescence with ASD [104], there is justified optimism regarding the potential for oxytocin-based treatments for disorders linked to social deficits. Indeed, studies have found intranasal oxytocin to promote endogenous release [112] and to be an effective adjunct for psychosocial therapy [111]. Therefore, further basic and translational research into this pivotal neurohormone's roles in health and disease is critical.
